# Effects of maternal exposure to an adsorbed acellular diphtheria-tetanus-pertussis (reduced-dose) combined vaccine on prenatal and postnatal development in Sprague-Dawley rats: a preclinical study

**DOI:** 10.3389/fimmu.2026.1884369

**Published:** 2026-07-27

**Authors:** Wenlu Kong, Han Chu, Qin Gu, Yan Ma, Lukui Cai, Jingyan Li, Xiaoyu Wang, Qiuyan Ji, Jiana Wen, Na Gao, Guang Ji, Wenzhu Hu, Ting Zhao, Ling Ping, Yuting Fu, Ying Li, Tiannan Gong, Yingfu Jin, Yufan Yang, Yixian Fu, Xinhua Qin, Huimei Zheng, Jingsi Yang, Jiangli Liang

**Affiliations:** 1 Institute of Medical Biology, Chinese Academy of Medical Sciences & Peking Union Medical College, Kunming, China; 2 State Key Laboratory of Respiratory Health and Multimorbidity, Beijing, China; 3 Kunming Scientific and Technological Innovation Center for R&D and Industrialization of Vaccines Against Novel and Emerging Highly Pathogenic Pathogens, Kunming, China

**Keywords:** maternal antibody transfer, maternal immunization, reproductive and developmental toxicity, safety evaluation, Tdacp vaccine

## Abstract

**Introduction:**

The resurgence of pertussis poses a severe threat to infants under 3 months of age due to an inherent early-life “immunity gap.” While maternal immunization with the tetanus, diphtheria, and acellular pertussis (Tdap/Tdacp) vaccine effectively mitigates this risk globally, no such vaccine is currently licensed for maternal use in China. This preclinical study aimed to systematically evaluate the reproductive and developmental toxicity, as well as the immunogenicity, of an independently developed Tdacp candidate vaccine in Sprague-Dawley (SD) rats.

**Methods:**

Eighty-eight female SD rats were randomly assigned to four groups: negative control, adjuvant control, low-dose (0.25 ml/rat), and high-dose (0.5 ml/rat). The vaccine was administered via intramuscular injection at three time points to maximize exposure: one week prior to mating, on gestation day 6 (GD 6), and on lactation day 7 (LD 7). Systemic assessments included maternal general toxicity, reproductive performance, and embryo-fetal development. Additionally, postnatal survival, physical growth, and neuro-reflex developmental landmarks of F1 offspring were monitored. Antigen-specific antibody titers in both F0 dams and F1 offspring were quantified to evaluate immunogenicity and maternal antibodies transfer.

**Results:**

Repeated maternal exposure to the Tdacp vaccine elicited no treatment-related maternal systemic toxicity; body weight, food consumption, and gestation length remained comparable to controls. No adverse effects were observed on mating, fertility, or uterine implantation. Furthermore, the vaccine did not negatively impact F1 offspring survival, sex ratio, physical morphology, or the acquisition of neuro-reflex milestones. Immunologically, the vaccine induced robust and stable antibody responses against all specific antigens, including pertussis toxin (PT), filamentous hemagglutinin (FHA), pertactin (PRN), diphtheria toxoid (DT), and tetanus toxoid (TT), in F0 dams. Crucially, high titers of these antigen-specific antibodies were detected in the F1 offspring, demonstrating successful passive transfer of maternal antibodies to the offspring.

**Conclusion:**

The investigational Tdacp vaccine demonstrated a highly favorable safety profile with no evidence of maternal, reproductive, or developmental toxicity. Coupled with its robust immunogenicity and efficient maternal antibody transfer, these findings provide pivotal preclinical validation to support advancing the vaccine into human clinical trials for maternal immunization.

## 1 Introduction

Pertussis, or whooping cough, is a highly contagious acute respiratory infectious disease caused by Bordetella pertussis, posing a severe threat to the health of infants and young children. Following the global implementation of whole-cell pertussis (wP) vaccines in the mid-20th century and their subsequent transition to acellular pertussis (aP) vaccines, the overall incidence of pertussis declined significantly (1, 2). However, over the past two decades, several developed nations—including Australia, the United States, and the United Kingdom—have reported a substantial rebound in incidence, a phenomenon recognized as “pertussis resurgence” (3, 4).

This resurgence has become particularly pronounced in China in recent years. Data from the Chinese Center for Disease Control and Prevention (China CDC) revealed that 476,690 pertussis cases and 30 deaths were reported nationwide in 2024 (5). The incidence rate surged from 2.91 per 100,000 in 2023 to 33.86 per 100,000—an approximately 12-fold increase (5–7). This shift indicates that pertussis in China has transitioned from a long-term low-prevalence state to a phase of rapid rebound, significantly elevating public health risks. Epidemiological research consistently demonstrates that severe and fatal cases are predominantly concentrated among infants under 3 months of age who have not yet completed their primary immunization series (8, 9); this population remains the core group bearing the highest disease burden.

Because the neonatal immune system is immature and the first dose of the diphtheria-tetanus-acellular pertussis (DTaP) vaccine is typically not administered until 2–3 months of age, an inherent “immunity gap” exists. This leaves infants at extremely high risk of infection and severe disease during their earliest stages of life. The “cocooning strategy”—which aims to protect neonates indirectly by vaccinating family members and close contacts—was initially proposed as a solution. However, its practical implementation has been hindered by insufficient coverage and the indirect, delayed nature of the immunity provided. Consequently, numerous studies have confirmed that the cocooning strategy is inadequate for effectively interrupting pertussis transmission to vulnerable infants (10).

In response to these real-world challenges, several nations have pivoted their prevention strategies toward booster immunization for adolescents, adults, and pregnant women. The Advisory Committee on Immunization Practices (ACIP) in the United States recommends the administration of the Tdacp vaccine for individuals aged 19 years and older, with pregnant women identified as a priority group (11). Since 2011, the ACIP has issued national recommendations for vaccinating pregnant women who had not previously received Tdap at or after 20 weeks of gestation (12). In 2012, these guidelines were further refined to explicitly recommend Tdap administration during every pregnancy, with the optimal window identified between 27 and 36 weeks of gestation (13). Extensive real-world studies and systematic reviews have demonstrated that maternal Tdap immunization can prevent approximately 70%–90% of infant pertussis cases and reduce the risk of pertussis-related hospitalization in infants under 3 months by as much as 90.5% (14).

Currently, most developed countries in Europe and North America have integrated Tdap vaccine into routine booster and maternal immunization programs. By elevating maternal antibody titers and facilitating maternal antibody transfer passive transfer, these programs provide effective immune protection during the critical first months of a neonate’s life, progressively establishing a life-course immunization strategy covering infants, adolescents, adults, and pregnant women (15–17).

In contrast, no Tdap vaccine specifically indicated for adult or maternal use is currently licensed in China. In the context of the rapidly rising incidence of pertussis, this vaccine accessibility gap has become a significant constraint in neonatal pertussis control, creating an urgent clinical and public health need. Given this backdrop, advancing the development of domestic Tdap vaccines is of profound public health significance and possesses substantial industrial potential. The investigational Tdacp vaccine evaluated in this study was designed based on the antigen composition used in internationally licensed reduced-antigen-content Tdap vaccines, with reduced amounts of diphtheria toxoid and pertussis antigens while maintaining a full tetanus toxoid dose. Such formulations have been widely adopted for booster and maternal immunization strategies because they provide adequate immunogenicity with improved reactogenicity profiles.

According to relevant technical guidelines, systemic evaluation of reproductive and developmental toxicity and immunogenicity in animal models during the perinatal period (gestation and lactation) is a mandatory prerequisite for clinical trials of vaccines intended for women of childbearing age and pregnant populations. Therefore, the study utilized an independently developed Tetanus, Diphtheria and Acellular Component Pertussis Vaccine Adsorbed (Reduced Antigens Content) (Tdacp) administered to rats during the perinatal period. This study aimed to systematically evaluate its reproductive and developmental safety profile and immunogenicity, thereby providing critical experimental evidence to fill the gap in safety data for maternal pertussis vaccines in China and support subsequent clinical research and regulatory decision-making.

## 2 Article types

Original Research.

## 3 Materials and methods

### 3.1 Test articles and control

The test article, adsorbed component acellular pertussis (reduced dose) combined vaccine (Tdacp; lot number: 20240301), was independently developed and manufactured by the Institute of Medical Biology, Chinese Academy of Medical Sciences (IMBCAMS). The formulation of one full human clinical dose (0.5 mL) consists of diphtheria toxoid (2 Lf), tetanus toxoid (2.5 Lf), pertussis toxin (8 μg), filamentous hemagglutinin (8 μg), and pertactin (2.5 μg), with 1.2 mg of aluminum hydroxide used as an adjuvant. The aluminum hydroxide adjuvant (adjuvant control) was also prepared by IMBCAMS. The 0.9% sodium chloride injection (negative control/vehicle) was manufactured by Shandong Qidu Pharmaceutical Co., Ltd.

### 3.2 Animals and husbandry

A total of 148 sexually mature SPF SD rats (60 males and 88 females) were provided by Beijing Vital River Laboratory Animal Technology Co., Ltd. [License No. SCXK (Jing) 2021-0006]. At the start of the study, male body weights ranged from 232.0 g to 272.3 g, and female weights ranged from 181.0 g to 224.1 g (Certificate Nos. №.110011241105141463 and №.110011241103180462). All animals were housed in a barrier facility maintained at 20–26 °C and 40–70% relative humidity, with ad libitum access to food and water. Following a 3-day quarantine and a 7-day acclimatization period, clinical evaluations including appearance, behavior, respiration, and palpation were performed. The study protocol was approved by the Institutional Animal Care and Use Committee (IACUC) of Shandong Xinbo Pharmaceutical Research Co., Ltd. (Approval No. XB-IACUC-20241160).

### 3.3 Experimental design

Eighty-eight sexually mature females were randomly assigned into four groups (n = 22/group) based on body weight using TOXSTAT 2006 software: negative control (Vehicle), adjuvant control, low-dose (L), and high-dose (H) groups. Males were used exclusively for mating and were not assigned to treatment groups. Females were co-housed 1:1 with males. The following morning, females were checked for the presence of a vaginal plug or a sperm-positive vaginal smear, the day of which was designated as GD 0.

**Figure 1:**
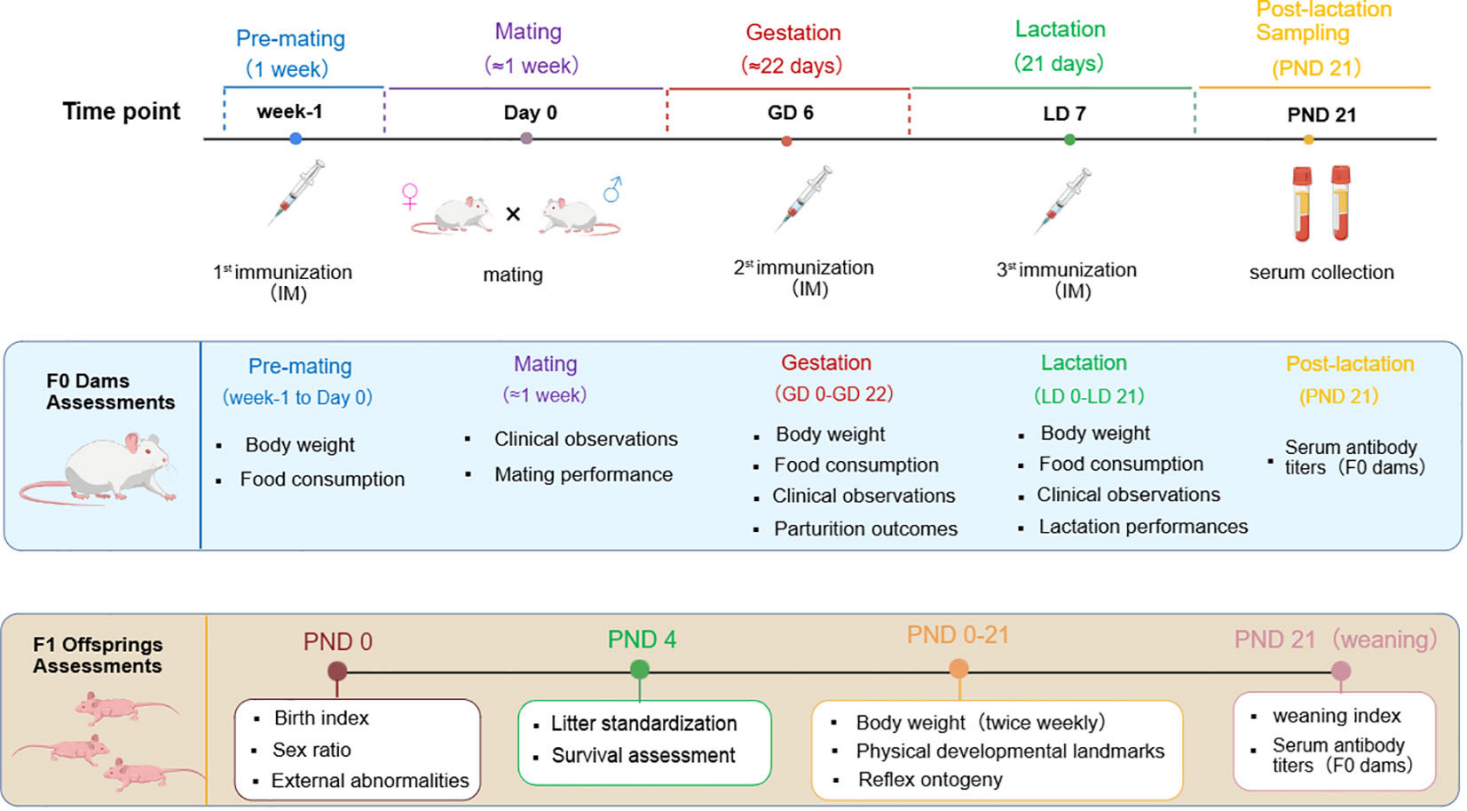
Experimental design and timeline of the developmental and reproductive toxicity (DART) study in Sprague–Dawley rats. Female rats were assigned to four groups: vehicle control, adjuvant control, low-dose Tdacp (0.25 mL/dose), and high-dose Tdacp (0.5 mL/dose). Animals received three intramuscular immunizations at one week before mating, gestation day (GD) 6, and lactation day (LD) 7. Following mating, dams were monitored throughout gestation and lactation for clinical signs, body weight, food consumption, reproductive performance, and parturition outcomes. F1 offspring were evaluated for postnatal survival, body weight gain, external abnormalities, physical developmental landmarks, and reflex ontogeny. Litters were standardized on postnatal day (PND) 4 and maintained until weaning on PND 21. Serum samples from F0 dams and F1 offspring were collected at PND 21 for antigen-specific IgG antibody analysis. Created with Figdraw.

Test articles were administered via intramuscular (IM) injection into the medial calf muscle at an angle of approximately 35°. The injection volume was 0.5 ml/rat for the Vehicle, adjuvant control, and H-dose groups (multi-site injection in both hind limbs, 0.25 ml/site) and 0.25 ml/rat for the L-dose group (alternating between hind limbs, 0.25 ml/site). Each female received three doses: one week prior to mating, on GD 6, and on LD 7.The overall study design and immunization schedule are summarized in **Figure 1**.

### 3.4 Observations and measurements

#### 3.4.1 Clinical observations

Animals were monitored once daily during acclimatization and twice daily (a.m. and p.m.) during the treatment period, with an additional observation within 1 hour post-dose. From GD 20, dams were monitored twice daily for 5 consecutive days for signs of abortion, premature delivery, or abnormal parturition (e.g., dystocia, delayed or incomplete delivery). Injection sites were inspected daily for local reactions.

#### 3.4.2 Body weight and food consumption

Measurements were taken twice weekly during the pre-mating period. During gestation, maternal body weight and 24-h food consumption were recorded every 3 days until GD 20. During the lactation period, measurements for F0 dams and F1 offspring were recorded on LD/PND 0, 4, 7, 10, 14, 17, and 21.

#### 3.4.3 F1 offspring evaluation

The day of birth was designated as postnatal day 0 (PND 0). Pups were assessed for survival, external morphology, and sex. Daily observations included physical development (pinna detachment, incisor eruption, ear opening, abdominal hair growth, eye opening) and reflex development (surface righting, cliff avoidance, tactile positioning, negative geotaxis, air righting, visual positioning). The time required for all pups in a litter to reach these milestones was recorded. Birth survival and weaning rates were calculated. On PND 4, litters were standardized to eight pups per litter (preferably four males and four females). When the litter size exceeded eight pups, surplus pups were selected randomly within each sex whenever possible and euthanized by CO2 inhalation. This procedure was performed to minimize selection bias and maintain consistency among litters.

#### 3.4.4 Terminal procedures and necropsy

On LD/PND 21, F0 dams and F1 offspring were euthanized by exsanguination from the abdominal aorta/vena cava under CO2 anesthesia. A gross necropsy of the thoracic and abdominal organs was performed, and the number of uterine implantation sites was recorded. Blood samples (1.5 ml/dam; 1.5 ml/litter for F1) were collected from the first 8 dams to deliver in each group (excluding the adjuvant control).Because aluminum hydroxide alone does not contain vaccine antigens and is not expected to induce antigen-specific antibodies, immunogenicity assessments were limited to the vehicle and Tdacp-treated groups. Serum IgG antibody levels against diphtheria, tetanus, and pertussis were quantified using an in-house indirect enzyme-linked immunosorbent assay (ELISA) developed by the Institute of IMBCAMS. Briefly, 96-well microplates were coated overnight at 4°C with purified PT, FHA, PRN, DT, or TT antigens (3–5 μg/mL). After blocking with 3% bovine serum albumin(BSA), serial two-fold dilutions of serum samples were added and incubated for 1 h at 37°C. To ensure accurate endpoint determination, serum samples from dams and offspring were diluted appropriately according to their expected antibody levels and analyzed using the same serial two-fold dilution procedure. Antibody titers were expressed as endpoint titers and were therefore independent of the initial dilution factor. Bound antibodies were detected using horseradish peroxidase-conjugated goat anti-rat IgG, followed by tetramethylbenzidine substrate development. Absorbance was measured at 450 nm. Individual antibody titers were converted to log2 values, and group geometric mean titers (GMTs) were calculated and used for graphical presentation and statistical analysis. The lower limit of detection (LLOD) of the assay was defined as a titer of 1:400. Samples with antibody titers below this limit were assigned a value of 400 for subsequent analysis and graphical presentation.

### 3.5 Statistical analysis

Data were tabulated using Microsoft Excel and analyzed using TOXSTAT 2006 statistical software.

For qualitative data (e.g., mortality, incidences of premature delivery, abortion, dystocia, delayed delivery, incomplete delivery, clinical signs, conception rate, and pregnancy rate), statistical comparisons were performed using Fisher’s exact test.

For quantitative data (e.g., F0 maternal body weights and food consumption during pre-mating, gestation, and lactation periods; F1 offspring developmental milestones, reflex development, body weight of surviving pups, external abnormality rates, birth survival rates, and weaning rates), the data were first evaluated for homogeneity of variance using Bartlett’s test. If the variance was homogeneous (p ≥ 0.05), a one-way analysis of variance (ANOVA) was performed. If Bartlett’s test indicated significant heterogeneity (p < 0.05), the Kruskal-Wallis test was utilized.

Following a significant ANOVA or Kruskal-Wallis test (p ≤ 0.05), *post-hoc* multiple comparisons were conducted using Dunnett’s parametric test or Dunnett’s non-parametric test, respectively. If the initial test (ANOVA or Kruskal-Wallis) was not significant (p > 0.05), no further analysis was performed. The sex ratio of F1 offspring was analyzed using the Pearson Chi-square test.

## 4 Results

### 4.1 General toxicity in F0 maternal rats

**Figure 2:**
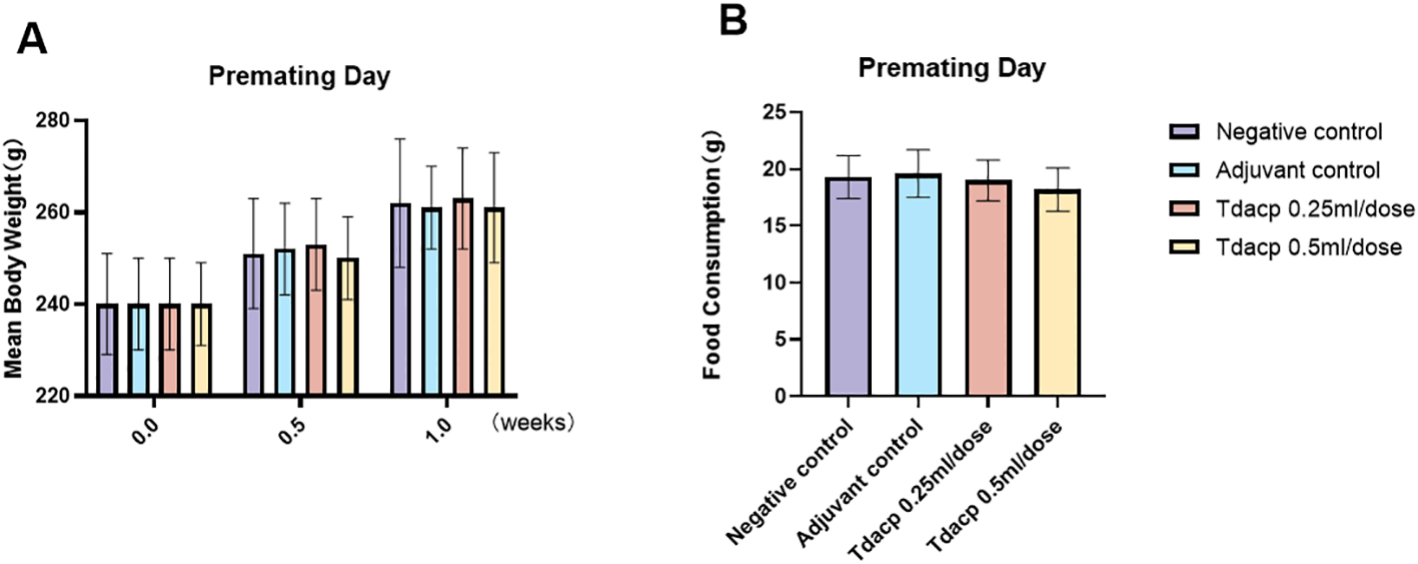
Body weight and food consumption of F0 female rats during the premating period. **(A)** Mean body weight during the premating period; **(B)** food consumption during the premating period. Body weight was measured individually and summarized for each treatment group. Food consumption was determined on a cage basis and expressed as grams per animal per day (g/animal/day). Values for food consumption represent the mean ± SD of cage means (n = 8 cages per group). Daily food consumption was calculated from the difference between feed supplied and residual feed and normalized to the number of animals housed per cage and the duration of the measurement interval. Data are presented as mean ± SD.

**Figure 3:**
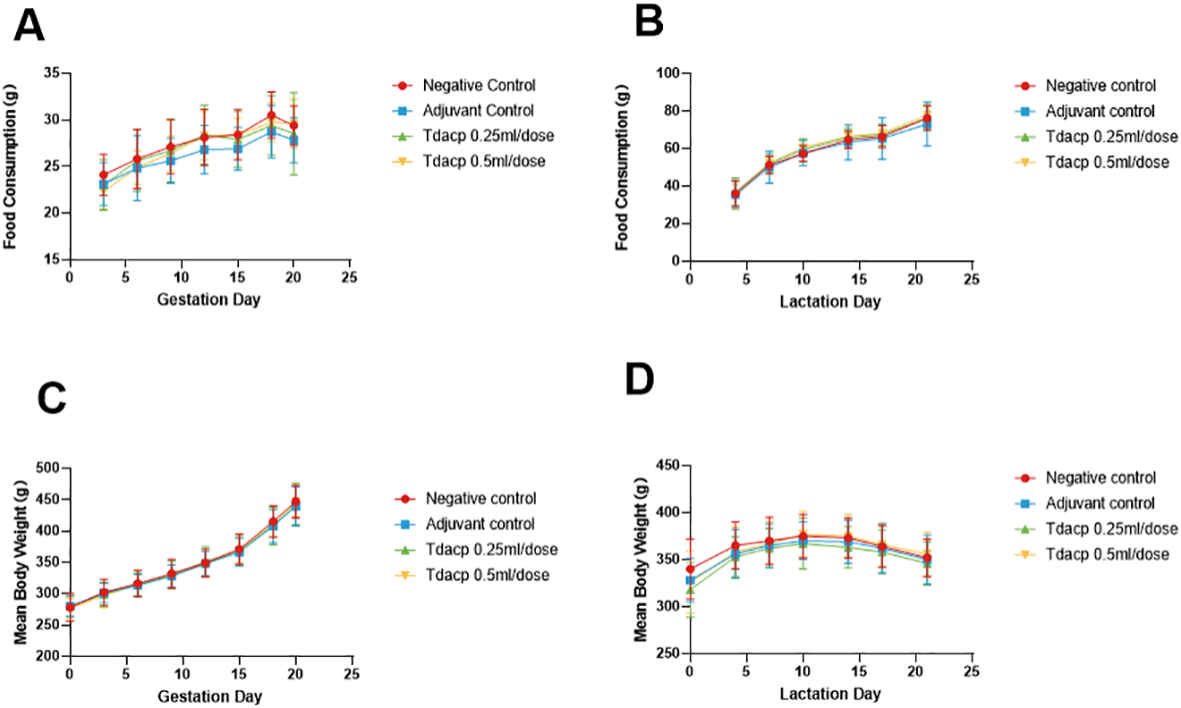
Maternal body weight and food consumption during gestation and lactation in F0 dams. **(A)** maternal food consumption during gestation; **(B)**maternal food consumption during lactation; **(C)** maternal body weight during gestation; and **(D)** maternal body weight during lactation. Body weight and food consumption were measured individually throughout the gestation and lactation periods. Food consumption was expressed as grams per animal per day (g/animal/day). Data are presented as mean ± SD. Statistical comparisons were performed using one-way ANOVA followed by Dunnett’s test. No treatment-related differences were observed among groups.

Administration of the Tdacp test vaccine had no toxicologically significant effects on the body weight or food consumption of F0 dams throughout the study period. During the premating period, body weight and food consumption remained comparable among all groups (**Figures 2A, B**). Similarly, both body weight and food intake increased progressively during gestation and lactation without treatment-related differences between groups (**Figures 3A–D**).

### 4.2 Maternal fertility and embryo-fetal development

No incidences of premature delivery, abortion, delayed delivery, or incomplete delivery were observed in any group. Animals in the negative control and adjuvant control groups showed no remarkable abnormalities (**Table 1**). In the H-dose group, one instance of pseudopregnancy was recorded. In the L-dose group, one maternal death occurred; gross necropsy revealed two dead fetuses and three retained placentas in the uterus. This death was attributed to dystocia and was considered unrelated to vaccine administration.

**Table 1:** |Mating performance and fertility outcomes in the maternal generation.

Parameters	Negative control (0.5 ml/rat)		Adjuvant control (0.5 ml/rat)		L-dose (0.25 ml/rat)		H-dose (0.5 ml/rat)	
	Frequency (n)	Incidence (%)	Frequency (n)	Incidence (%)	Frequency (n)	Incidence (%)	Frequency (n)	Incidence (%)
Mated females	22/22	100	22/22	100	22/22	100	22/22	100
Pseudopregnancy	0/22	0	0/22	0	0/22	0	1/22	4.5
Pregnancy	22/22	100	22/22	100	22/22	100	21/22	95.5
Pre-mating mortality	0/22	0	0/22	0	0/22	0	0/22	0
Parturient dams	22/22	100	22/22	100	22/22	100	21/21	100
Maternal mortality (during gestation)	0/22	0	0/22	0	1/22	4.5	0/21	0
Maternal mortality (during lactation)	0/22	0	0/22	0	0/22	0	0/21	0
Premature delivery	0/22	0	0/22	0	0/22	0	0/21	0
Abortion	0/22	0	0/22	0	0/22	0	0/21	0
Dystocia	0/22	0	0/22	0	1/22	4.5	0/21	0
Delayed delivery	0/22	0	0/22	0	0/22	0	0/21	0
Incomplete delivery	0/22	0	0/22	0	0/22	0	0/21	0

Data are presented as frequency and incidence (%). Comparisons with the negative control group were performed using Fisher’s exact test.

During the study, no treatment-related clinical signs were observed in the adjuvant control, L-dose, or H-dose groups. Parameters such as uterine implantation sites and gestation length were unaffected by Tdacp administration (**Table 2**). Although a statistically significant difference was observed in the span of gestation length in the L-dose group compared to the negative control, all values remained within the historical control range for SD rats reported by our testing facility (gestation length: 21–23 days) and were considered biological variation unrelated to the test article.

**Table 2:** Maternal clinical observations following administration, including duration of gestation and number of implantation sites.

Examination parameters	Negative control (0.5 ml/rat)	Adjuvant control (0.5 ml/rat)	L-dose (0.25 ml/rat)	H-dose (0.5 ml/rat)
n	22	22	21	21
Abnormal clinical signs	0	0	0	0
Gestation length range	21-22	22-23	22-23	22-23
Gestation length	22.0 ± 0.2	22.1 ± 0.3	22.3 ± 0.5**(P = 0.0061)	22.1 ± 0.4
Number of uterine implantation sites	16 ± 3	16 ± 3	17 ± 1	17 ± 2

Data are presented as mean ± SD. Comparisons with the negative control group were performed using one-way ANOVA followed by Dunnett’s test.

### 4.3 Postnatal developmental toxicity

Growth and developmental data for F1 offspring, including weaning rate, sex ratio, and external morphology, showed no statistically significant differences compared to the negative control group (**Table 3**). The mean birth survival rate was 98.8% in the negative control group and 96.3% in the H-dose group. Although this difference reached statistical significance, the value observed in the H-dose group remained within the historical control range and was not considered treatment-related. No other remarkable abnormalities were noted.

**Table 3:** Postnatal viability, sex distribution, and incidence of external anomalies in the F1 Offspring.

Examination parameters		Negative control (0.5 ml/rat)	Adjuvant control (0.5 ml/rat)	L-dose (0.25 ml/rat)	H-dose (0.5 ml/rat)
Number of litters examined (n)		22	22	21	21
Number of live pups at birth (PND0)		311	314	318	317
Number of dead pups at birth(PND0)		4	4	11	8
Number of pups surviving at PND 4		308	312	304	305
Number of pups retained at PND 4 (Standardization)		175	169	168	168
Number of pups surviving at PND 21 (Weaning)		175	168	168	168
Birth index (%)	(MEAN±SD)	98.8 ± 3.2	99.4 ± 1.9	95.7 ± 8.0	96.3 ± 3.8*(P = 0.0495)
Weaning index (%)	(MEAN±SD)	100.0 ± 0.0	99.4 ± 2.7	100.0 ± 0.0	100.0 ± 0.0
Sex ratio (Male:Female)		1.1(149:162)	1.2 (146:168)	1.1 (154:164)	0.9 (166:151)
Total number of pups examined		311	314	318	317
Number of pups with external abnormalities		0	0	0	0

Birth index and weaning index are expressed as mean ± SD and were analyzed by one-way ANOVA followed by Dunnett’s test. Sex ratio was analyzed using Pearson’s chi-square test. Incidences of external abnormalities were compared using Fisher’s exact test.

Birth index (%) = (Number of live pups at birth/Total number of pups delivered) ×100.

Weaning index (%) = (Number of pups surviving to PND 21/Number of pups retained after litter standardization on PND 4) ×100.

### 4.4 Growth and developmental landmarks of F1 offspring

**Figure 4:**
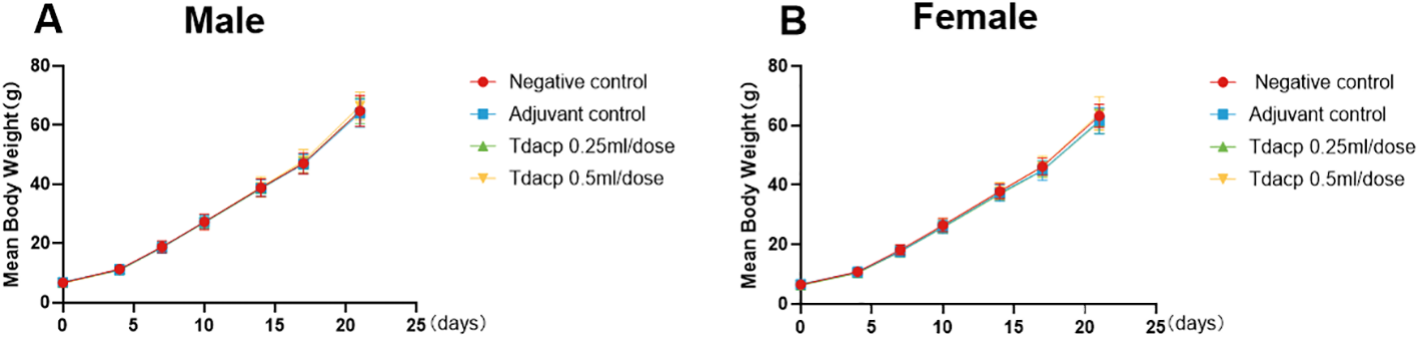
Postnatal body weight trajectories of F1 offspring. **(A)** Male offspring; **(B)** Female offspring. Body weights were recorded on postnatal day (PND) 0, 4, 7, 10, 14, 17, and 21. Data are presented as mean ± SD. Statistical comparisons were performed using one-way ANOVA followed by Dunnett’s test. No statistically significant differences in body weight were observed between the Tdacp-treated groups and the negative control group throughout the observation period.

Body weights of both male and female F1 offspring increased progressively throughout the study, with no statistically significant differences observed compared to the negative control group(**Figures 4A, B**). To evaluate the potential impact of the Tdacp test vaccine on the development of F1 offspring, physical and reflex developmental milestones were monitored. The mean age (PND) of attainment for all developmental landmarks in the adjuvant control, low-dose, and high-dose groups showed no statistically significant differences relative to the negative control group (**Table 4**).

**Table 4:** Evaluation of physical developmental landmarks and reflex ontogeny in F1 offspring.

Examination parameters	Negative control (0.5 ml/rat)	Adjuvant control (0.5 ml/rat)	L-dose (0.25 ml/rat)	H-dose (0.5 ml/rat)
Number of litters examined (n)	22	22	21	21
Physical development (Landmarks)(PND, mean ± SD)				
Pinna detachment	3 ± 0	3 ± 0	3 ± 0	3 ± 0
Lower incisor eruption	12 ± 1	12 ± 1	12 ± 0	12 ± 1
Ear canal opening	13 ± 1	13 ± 1	13 ± 1	13 ± 1
Abdominal hair growth	14 ± 1	14 ± 1	14 ± 0	14 ± 0
Eye opening	14 ± 1	14 ± 1	14 ± 0	14 ± 0
Reflex development(PND, mean ± SD)				
Surface righting (reflex)	4 ± 1	4 ± 1	4 ± 1	4 ± 1
Cliff avoidance	6 ± 1	7 ± 1	6 ± 1	6 ± 1
Whisker orienting	10 ± 1	10 ± 1	10 ± 1	10 ± 1
Negative geotaxis	11 ± 1	11 ± 1	11 ± 1	11 ± 1
Air righting (reflex)	16 ± 1	16 ± 1	16 ± 1	16 ± 1
Visual positioning	16 ± 1	16 ± 1	16 ± 1	16 ± 1

Data are expressed as mean ± SD of postnatal day (PND) at which all pups within a litter attained each developmental landmark. Comparisons with the negative control group were performed using one-way ANOVA followed by Dunnett’s test.

### 4.5 Immunogenicity evaluation

#### 4.5.1 Antibody levels in F0 dams

**Figure 5:**
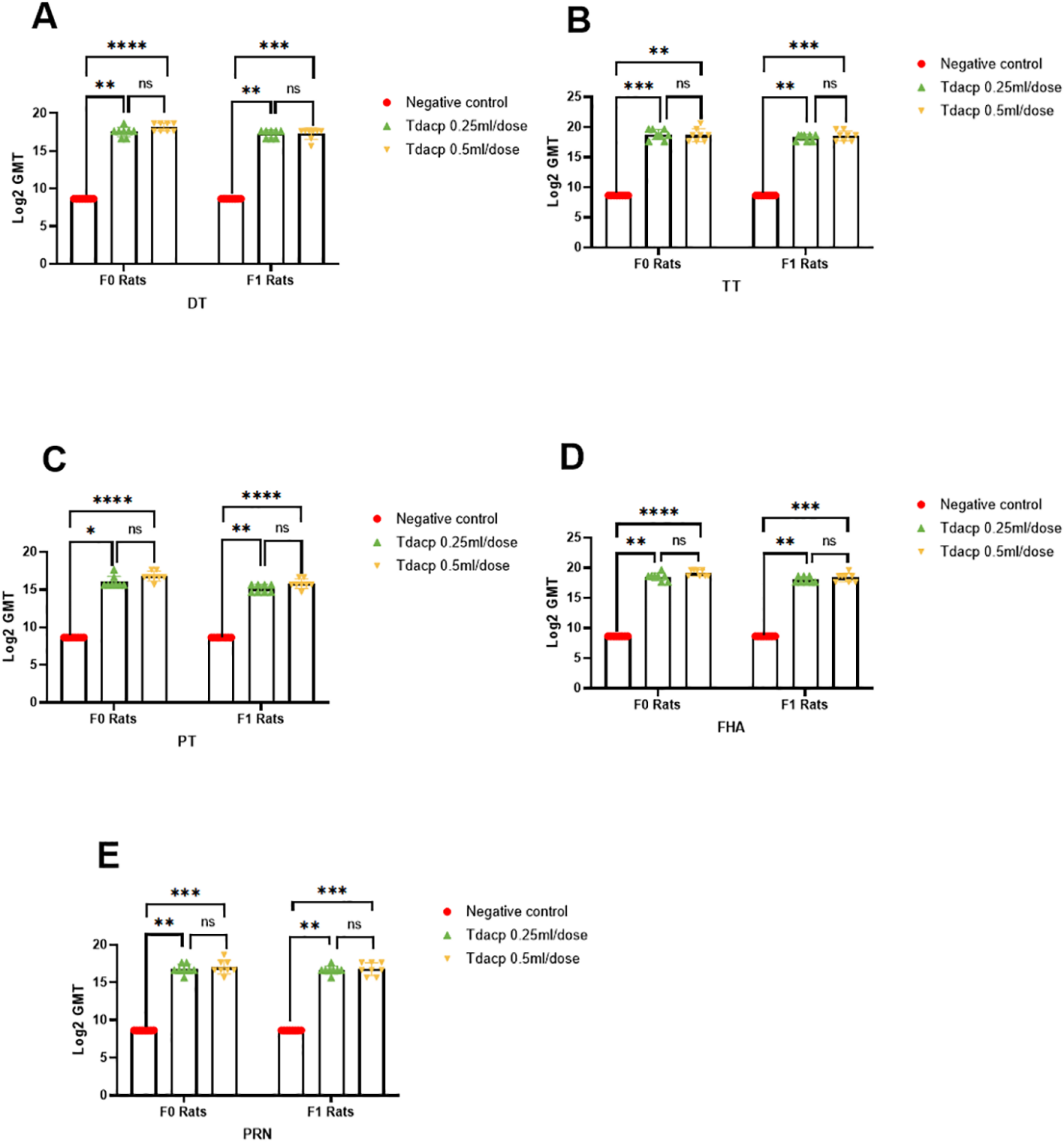
Antigen-specific IgG antibody responses in F0 dams and F1 offspring at postnatal day (PND) 21. **(A)** Anti-diphtheria toxoid (DT) IgG antibody responses; **(B)** anti-tetanus toxoid (TT) IgG antibody responses; **(C)** anti-pertussis toxin (PT) IgG antibody responses; **(D)** anti-filamentous hemagglutinin (FHA) IgG antibody responses; and **(E)** anti-pertactin (PRN) IgG antibody responses. Serum samples collected from F0 dams and F1 offspring were analyzed by indirect ELISA. Data are presented as log₂-transformed geometric mean titers (GMTs). Statistical comparisons were performed separately within F0 dams and F1 offspring using one-way ANOVA followed by multiple comparison tests. Samples with antibody titers below the lower limit of detection (LLOD; 1:400) were assigned a value of 400 for analysis and graphical presentation. The dashed line indicates the LLOD. Statistical significance is indicated as follows: ns, not significant; P < 0.05; P < 0.01; P < 0.001.

Antibody titers against all vaccine components (diphtheria, tetanus, and pertussis) were negative in the negative control group. In contrast, the low-dose and high-dose groups were seropositive for all components. Although numerical variations in antibody titers were observed between the low- and high-dose groups for DT, PT, FHA, PRN, and TT, these differences did not reach statistical significance (P > 0.05) (**Figures 5A–E**).

#### 4.5.2 Antibody levels in F1 offspring

All antibody titers against the vaccine components were negative in the F1 offspring of the negative control group. In the low-dose and high-dose groups, antibody titers for all components were positive, suggesting successful maternal antibody transfer to the offspring. (**Figures 5A–E**).

## 5 Discussion

The investigational Tdacp candidate vaccine evaluated in this study is formulated with a full dose of TT and reduced doses of DT and acellular pertussis antigens, specifically PT, FHA, and PRN, adsorbed to an aluminum hydroxide adjuvant. The antigen composition of the investigational Tdacp vaccine is broadly comparable to internationally licensed reduced-antigen-content Tdap vaccines used for maternal immunization, although species-specific differences preclude direct extrapolation of antigen doses on a per-body-weight basis. The experimental design and implementation were conducted in strict compliance with the ICH S5(R3) guideline, the Technical Guidelines for Reproductive Toxicity Studies of Drugs (2006), and the General Principles for Technical Review of Preclinical Safety Evaluation of Preventive Biological Products (2005) (18–20). The primary objective was to comprehensively assess the potential maternal and developmental toxicity of Tdacp following administration during gestation and lactation, thereby establishing a critical safety profile to support its clinical application in pregnant women.

This study systematically evaluated both embryo-fetal development (EFD) and pre- and postnatal development (PPND). SD rats were selected as the experimental model, as they are the preferred species recommended by regulatory authorities for reproductive toxicity testing, owing to their extensive historical control data and well-characterized reproductive physiology (21).

Although a single dose of the Tdacp vaccine is standard clinical practice during the second or third trimester of pregnancy, a modified multi-dose regimen was utilized in this study to account for the significantly abbreviated gestation period of rats (approximately 21 days). In accordance with regulatory guidelines for developmental toxicity assessments, this three-dose schedule was strategically designed to maximize maternal and fetal exposure to both the vaccine antigens and the adjuvant system, rather than to replicate the human clinical schedule. Consequently, the primary objective of this study is the comprehensive evaluation of perinatal safety—including specific considerations for adjuvant-related toxicity—rather than the optimization of immunization schedules or the determination of the minimum dose required to elicit protective antibody responses. To elicit robust vaccine-induced immune responses and maintain stable antibody titers, the initial dose was administered one week prior to mating. Furthermore, in alignment with the regulatory principle that nonclinical safety studies should include at least one additional dose beyond the intended clinical regimen, a booster was administered on GD 6. A third dose was then administered on LD 7 to further evaluate the potential impact of vaccine-induced immunity on the offspring during the postnatal period.

Furthermore, Based on the standard human clinical dose (0.5 mL/dose), the subjects were assigned to two treatment groups: a low-dose group (0.25 mL/dose) and a high-dose group (0.5 mL/dose). The inclusion of both low- and high-dose groups represented relative exposure levels, rather than interpreted them as establishing cross-species dose equivalence. Both dosage groups exhibited high seroconversion rates and robust antibody responses, demonstrating that the Tdacp candidate vaccine possesses a favorable immunogenicity profile.

The results of the present study substantiate the favorable safety profile of the Tdacp vaccine in F0 maternal rats. Following repeated administration during the gestation and lactation periods, maternal parameters—including body weight gain, food consumption, and gestation length—remained comparable to those of the concurrent vehicle control group, indicating no vaccine-related systemic toxicity.

Regarding reproductive outcomes, no adverse effects were observed on mating behavior, estrous cyclicity, or fertility. The mean number of uterine implantation sites and parameters related to parturition and lactation were unaffected by vaccination. Specific incidental findings were carefully adjudicated: the single maternal death in the low-dose group was attributed to dystocia (confirmed by gross necropsy revealing retained fetuses and placentas). As this was an isolated event not replicated in the high-dose group, it was deemed an incidental perinatal occurrence unrelated to the test article. Similarly, the single instance of pseudopregnancy in the high-dose group fell within the historical incidence range for SD rats and was considered toxicologically insignificant.

The assessment of offspring safety, a cornerstone of Developmental and reproductive toxicity (DART) testing, revealed no vaccine-induced developmental delays or neurotoxicity (22). F1 offspring across all groups exhibited comparable timing in achieving physical developmental landmarks (e.g., pinna detachment, eye opening) and neuro-reflex maturation (e.g., surface righting, cliff avoidance).

A specific statistical observation warrants clarification: while a statistically significant reduction in the birth survival rate was noted in the high-dose group (96.3%) compared to the control (98.8%, p < 0.05), statistical significance in this context does not equate to biological toxicity. The observed value remained well within the laboratory’s historical control range(95.0–100.0%), and considering the known natural fluctuations in perinatal survival of SD rats, along with the absence of a dose-response relationship or effects on weaning rates, this finding was interpreted as biological variation rather than a vaccine-mediated adverse effect (23, 24).

Beyond the safety evaluation, the study provided compelling evidence of the vaccine’s immunogenicity. Both low- and high-dose groups exhibited strong antibody responses against all antigenic components (PT, FHA, PRN, DT, and TT). Importantly, antigen-specific antibodies were detected in F1 offspring that had not been directly immunized. In rodents, maternally derived IgG can be transferred to offspring through both prenatal and postnatal pathways. Prenatal transfer occurs via the yolk-sac placenta, whereas postnatal transfer is mediated by FcRn-dependent uptake of milk-derived antibodies during lactation. Therefore, the antigen-specific antibodies detected in F1 offspring at PND 21 likely reflect the combined contribution of these two physiological pathways and indicate successful maternal antibody transfer. From a translational perspective, maternally derived antibodies induced by Tdacp vaccination may provide passive protection during the vulnerable period before completion of the infant primary immunization series. This early protection is particularly important because young infants are at the highest risk of severe pertussis-related morbidity and mortality. Although previous clinical studies have suggested that maternally derived antibodies may influence the magnitude of certain infant vaccine responses, the overall benefits of maternal immunization in protecting infants during early life are considered to outweigh these effects.

This mechanism of passive immunization validates the scientific rationale behind the ACIP’s recommendation for maternal vaccination: leveraging maternal antibodies to bridge the infant’s “immunity gap” prior to the maturation of their own immune system (13). Notably, the lack of a significant difference in antibody induction levels between the L-dose and H-dose groups suggests that the reduced-dose formulation is sufficient to elicit a robust immune response while maintaining an optimal safety profile, providing critical data to support dose selection for subsequent clinical trials.

Several limitations of this study should be acknowledged. Antibody titers were assessed only at PND 21, which precluded characterization of the kinetics of the immune response. In addition, because antibody levels at birth and antibodies in maternal milk were not evaluated, the relative contributions of prenatal and postnatal transfer could not be distinguished. Therefore, the presence of antigen-specific antibodies in F1 offspring should be interpreted as evidence of successful maternal antibody transfer rather than proof of a specific transplacental mechanism. Further studies are warranted to clarify the respective roles of placental and lactational antibody transfer.

## 6 Conclusion

Collectively, this study substantiates the favorable safety profile of the Tdacp vaccine in a PPND setting in SD rats. Within the intended clinical dose range, no treatment-related adverse effects were observed regarding maternal pregnancy, embryo-fetal development, postnatal growth, neuro-reflex maturation, or reproductive performance. Concomitantly, the vaccine elicited robust maternal antibody responses that were efficiently transferred to the offspring, validating the mechanism of passive neonatal protection. These data provide a pivotal scientific foundation for advancing Tdacp into clinical trials for pregnant women and other target populations, underscoring its potential to address the critical gap in maternal immunization and optimize the life-course immunization strategy for pertussis in China.

## Data Availability

The original contributions presented in the study are included in the article/supplementary material. Further inquiries can be directed to the corresponding author.
